# 1-[2-(3,5-Difluoro­benz­yloxy)phen­yl]ethanone

**DOI:** 10.1107/S160053681003120X

**Published:** 2010-08-11

**Authors:** Ya-Tuan Ma, Xin-Wei Shi, Qi Shuai, Jin-Ming Gao

**Affiliations:** a College of Science, Northwest A&F University, Yangling 712100, People’s Republic of China

## Abstract

In the title compound, C_15_H_12_F_2_O_2_, the dihedral angle between the aromatic rings is 70.43 (4)°. The crystal packing exhibits no significantly short inter­molecular contacts.

## Related literature

For background to the Williamson reaction in organic synthesis, see: Dermer (1934 ▶). For a related structure, see: Ma *et al.* (2010 ▶).
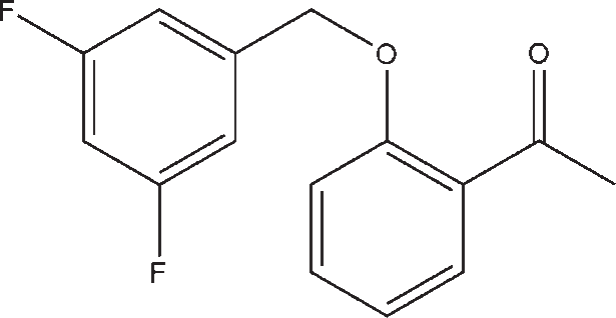


## Experimental

### 

#### Crystal data

C_15_H_12_F_2_O_2_
*M* *_r_* = 262.25
Triclinic, 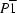

*a* = 7.2808 (7) Å
*b* = 7.9734 (8) Å
*c* = 11.6466 (12) Å
α = 91.587 (1)°
β = 106.559 (2)°
γ = 95.343 (1)°
*V* = 644.22 (11) Å^3^
*Z* = 2
Mo *K*α radiation
μ = 0.11 mm^−1^
*T* = 298 K
0.42 × 0.38 × 0.20 mm

#### Data collection

Bruker SMART CD area-detector diffractometer
Absorption correction: multi-scan (*SADABS*; Sheldrick, 1996 ▶) *T*_min_ = 0.956, *T*_max_ = 0.979
3373 measured reflections
2239 independent reflections
1402 reflections with *I* > 2σ(*I*)
*R*_int_ = 0.014

#### Refinement

*R*[*F* ^2^ > 2σ(*F* ^2^)] = 0.049
*wR*(*F* ^2^) = 0.153
*S* = 1.03
2239 reflections
173 parameters
H-atom parameters constrained
Δρ_max_ = 0.21 e Å^−3^
Δρ_min_ = −0.20 e Å^−3^

### 

Data collection: *SMART* (Bruker, 1996 ▶); cell refinement: *SAINT* (Bruker, 1996 ▶); data reduction: *SAINT*; program(s) used to solve structure: *SHELXS97* (Sheldrick, 2008 ▶); program(s) used to refine structure: *SHELXL97* (Sheldrick, 2008 ▶); molecular graphics: *SHELXTL* (Sheldrick, 2008 ▶); software used to prepare material for publication: *SHELXTL*.

## Supplementary Material

Crystal structure: contains datablocks I, global. DOI: 10.1107/S160053681003120X/zs2054sup1.cif

Structure factors: contains datablocks I. DOI: 10.1107/S160053681003120X/zs2054Isup2.hkl

Additional supplementary materials:  crystallographic information; 3D view; checkCIF report

## Figures and Tables

**Fig. 1.**
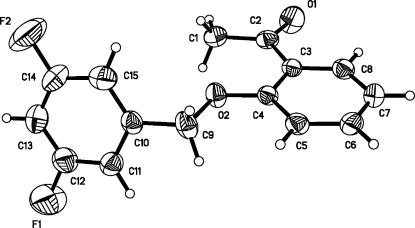
The molecular structure of (I) with atom labels, and displacement ellipsoids drawn at the 30% probability level.

## supplementary crystallographic information

### Comment

The Williamson reaction is a very useful transformation in organic synthesis since the products are of value in both industrial and academic applications. It usually involves the reaction of an alkali-metal salt of a hydroxy compound and an alkyl halide (Dermer, 1934). In the present paper, we present the structure of the title compound, C_15_H_12_F_2_O_2_ (I), which was synthesized by the reaction of 1-(2-hydroxyphenyl)ethanone, potassium carbonate and 3,5-difluorobenzyl bromide. We have previously reported the structure of a compound of this type (Ma *et al.*, 2010). In (I) (Fig. 1), the ethanone group is close to coplanar with the benzene ring [torsion angle C4–C3–C2–O1, 178.8 (2)°] while the dihedral angle between the aromatic rings is 70.43 (4)°. The crystal packing exhibits no significantly short intermolecular contacts.

### Experimental

1-(2-Hydroxyphenyl)ethanone (4 mmol), potassium carbonate (8 mmol), 3,5-difluorobenzyl bromide (4 mmol), and 40 ml acetone were mixed in a 100 ml flask. After 3 h stirring at 331 K, the crude product was obtained. Crystals of (I) were obtained by recrystallization from *n*-hexane/ethyl acetate.

### Refinement

The positions of all H atoms were determined geometrically and refined using a riding model with C—H = 0.93–0.97 Å and *U*_iso_(methyl H) = 1.5*U*_eq_(C) and 1.5*U*_eq_ for other H atoms.

### Crystal data

**Table:**

C_15_H_12_F_2_O_2_	*Z* = 2
*M**_r_* = 262.25	*F*(000) = 272
Triclinic, *P*1	*D*_x_ = 1.352 Mg m^−^^3^
Hall symbol: -P 1	Melting point = 347–348 K
*a* = 7.2808 (7) Å	Mo *K*α radiation, λ = 0.71073 Å
*b* = 7.9734 (8) Å	Cell parameters from 1136 reflections
*c* = 11.6466 (12) Å	θ = 2.9–24.8°
α = 91.587 (1)°	µ = 0.11 mm^−^^1^
β = 106.559 (2)°	*T* = 298 K
γ = 95.343 (1)°	Plate, colorless
*V* = 644.22 (11) Å^3^	0.42 × 0.38 × 0.20 mm

### Data collection

**Table:**

Bruker SMART CD area-detector diffractometer	2239 independent reflections
Radiation source: fine-focus sealed tube	1402 reflections with *I* > 2σ(*I*)
graphite	*R*_int_ = 0.014
φ and ω scans	θ_max_ = 25.0°, θ_min_ = 1.8°
Absorption correction: multi-scan (*SADABS*; Sheldrick, 1996)	*h* = −8→8
*T*_min_ = 0.956, *T*_max_ = 0.979	*k* = −9→5
3373 measured reflections	*l* = −13→13

### Refinement

**Table:**

Refinement on *F*^2^	Primary atom site location: structure-invariant direct methods
Least-squares matrix: full	Secondary atom site location: difference Fourier map
*R*[*F*^2^ > 2σ(*F*^2^)] = 0.049	Hydrogen site location: inferred from neighbouring sites
*wR*(*F*^2^) = 0.153	H-atom parameters constrained
*S* = 1.03	*w* = 1/[σ^2^(*F*_o_^2^) + (0.0702*P*)^2^ + 0.1465*P*] where *P* = (*F*_o_^2^ + 2*F*_c_^2^)/3
2239 reflections	(Δ/σ)_max_ = 0.001
173 parameters	Δρ_max_ = 0.21 e Å^−^^3^
0 restraints	Δρ_min_ = −0.20 e Å^−^^3^

### Special details

**Table:**

Geometry. All e.s.d.'s (except the e.s.d. in the dihedral angle between two l.s. planes) are estimated using the full covariance matrix. The cell e.s.d.'s are taken into account individually in the estimation of e.s.d.'s in distances, angles and torsion angles; correlations between e.s.d.'s in cell parameters are only used when they are defined by crystal symmetry. An approximate (isotropic) treatment of cell e.s.d.'s is used for estimating e.s.d.'s involving l.s. planes.
Refinement. Refinement of *F*^2^ against ALL reflections. The weighted *R*-factor *wR* and goodness of fit *S* are based on *F*^2^, conventional *R*-factors *R* are based on *F*, with *F* set to zero for negative *F*^2^. The threshold expression of *F*^2^ > σ(*F*^2^) is used only for calculating *R*-factors(gt) *etc*. and is not relevant to the choice of reflections for refinement. *R*-factors based on *F*^2^ are statistically about twice as large as those based on *F*, and *R*- factors based on ALL data will be even larger.

### Fractional atomic coordinates and isotropic or equivalent isotropic displacement parameters (Å^2^)

**Table:**

	*x*	*y*	*z*	*U*_iso_*/*U*_eq_	
F1	0.1438 (3)	0.8789 (4)	0.0404 (2)	0.1646 (11)	
F2	0.6758 (4)	0.6785 (4)	−0.0348 (2)	0.1880 (13)	
O1	0.7550 (3)	0.2045 (2)	0.54230 (18)	0.0964 (7)	
O2	0.7176 (2)	0.67975 (18)	0.41603 (13)	0.0629 (5)	
C1	0.6631 (4)	0.3400 (3)	0.3629 (2)	0.0627 (7)	
H1A	0.6321	0.2260	0.3292	0.094*	
H1B	0.7598	0.3965	0.3321	0.094*	
H1C	0.5495	0.3981	0.3416	0.094*	
C2	0.7371 (3)	0.3388 (3)	0.4951 (2)	0.0564 (6)	
C3	0.7914 (3)	0.4967 (3)	0.57422 (19)	0.0471 (5)	
C4	0.7837 (3)	0.6620 (3)	0.53600 (19)	0.0481 (5)	
C5	0.8396 (3)	0.7987 (3)	0.6196 (2)	0.0583 (6)	
H5	0.8355	0.9080	0.5938	0.070*	
C6	0.9010 (4)	0.7728 (3)	0.7398 (2)	0.0659 (7)	
H6	0.9379	0.8650	0.7950	0.079*	
C7	0.9086 (4)	0.6135 (3)	0.7795 (2)	0.0674 (7)	
H7	0.9501	0.5966	0.8612	0.081*	
C8	0.8540 (3)	0.4786 (3)	0.6971 (2)	0.0590 (6)	
H8	0.8592	0.3703	0.7247	0.071*	
C9	0.7050 (4)	0.8454 (3)	0.3733 (2)	0.0700 (7)	
H9A	0.8327	0.9048	0.3882	0.084*	
H9B	0.6320	0.9090	0.4138	0.084*	
C10	0.6061 (4)	0.8257 (3)	0.2421 (2)	0.0587 (6)	
C11	0.4210 (4)	0.8648 (3)	0.1996 (2)	0.0682 (7)	
H11	0.3585	0.9069	0.2518	0.082*	
C12	0.3289 (4)	0.8415 (4)	0.0801 (3)	0.0856 (9)	
C13	0.4081 (5)	0.7794 (4)	−0.0002 (3)	0.0902 (9)	
H13	0.3405	0.7632	−0.0811	0.108*	
C14	0.5903 (6)	0.7418 (5)	0.0421 (3)	0.0977 (10)	
C15	0.6927 (5)	0.7637 (4)	0.1624 (3)	0.0914 (9)	
H15	0.8189	0.7365	0.1887	0.110*	

### Atomic displacement parameters (Å^2^)

**Table:**

	*U*^11^	*U*^22^	*U*^33^	*U*^12^	*U*^13^	*U*^23^
F1	0.0955 (15)	0.243 (3)	0.1260 (18)	0.0644 (17)	−0.0240 (13)	−0.0392 (17)
F2	0.187 (3)	0.284 (4)	0.1253 (19)	0.063 (2)	0.0883 (18)	−0.035 (2)
O1	0.163 (2)	0.0435 (11)	0.0898 (14)	0.0206 (11)	0.0447 (13)	0.0108 (10)
O2	0.0905 (12)	0.0383 (9)	0.0520 (10)	0.0087 (8)	0.0073 (8)	0.0027 (7)
C1	0.0713 (16)	0.0452 (13)	0.0708 (16)	0.0032 (11)	0.0215 (13)	−0.0080 (11)
C2	0.0622 (15)	0.0418 (13)	0.0717 (16)	0.0098 (10)	0.0285 (12)	0.0048 (11)
C3	0.0466 (12)	0.0453 (12)	0.0519 (13)	0.0099 (9)	0.0164 (10)	0.0051 (10)
C4	0.0481 (12)	0.0447 (12)	0.0507 (13)	0.0090 (9)	0.0117 (10)	0.0024 (10)
C5	0.0618 (15)	0.0463 (13)	0.0625 (15)	0.0097 (11)	0.0103 (12)	−0.0028 (11)
C6	0.0668 (16)	0.0672 (17)	0.0589 (16)	0.0124 (12)	0.0101 (12)	−0.0132 (12)
C7	0.0713 (17)	0.0807 (19)	0.0501 (14)	0.0160 (14)	0.0148 (12)	0.0049 (13)
C8	0.0598 (14)	0.0578 (15)	0.0646 (16)	0.0149 (11)	0.0227 (12)	0.0135 (12)
C9	0.0921 (19)	0.0431 (13)	0.0622 (15)	0.0051 (12)	0.0025 (13)	0.0081 (11)
C10	0.0715 (16)	0.0443 (13)	0.0597 (15)	0.0077 (11)	0.0166 (12)	0.0110 (11)
C11	0.0706 (18)	0.0718 (17)	0.0618 (16)	0.0081 (13)	0.0189 (13)	−0.0016 (12)
C12	0.0740 (19)	0.099 (2)	0.075 (2)	0.0192 (16)	0.0052 (16)	−0.0020 (16)
C13	0.102 (2)	0.106 (2)	0.0591 (18)	0.0128 (19)	0.0171 (17)	0.0042 (16)
C14	0.117 (3)	0.117 (3)	0.076 (2)	0.022 (2)	0.052 (2)	−0.0050 (18)
C15	0.082 (2)	0.105 (2)	0.091 (2)	0.0324 (17)	0.0235 (17)	0.0040 (18)

### Geometric parameters (Å, °)

**Table:**

F1—C12	1.359 (3)	C6—H6	0.9300
F2—C14	1.340 (4)	C7—C8	1.372 (3)
O1—C2	1.218 (3)	C7—H7	0.9300
O2—C4	1.358 (2)	C8—H8	0.9300
O2—C9	1.426 (3)	C9—C10	1.489 (3)
C1—C2	1.480 (3)	C9—H9A	0.9700
C1—H1A	0.9600	C9—H9B	0.9700
C1—H1B	0.9600	C10—C11	1.365 (3)
C1—H1C	0.9600	C10—C15	1.368 (4)
C2—C3	1.491 (3)	C11—C12	1.361 (4)
C3—C8	1.389 (3)	C11—H11	0.9300
C3—C4	1.404 (3)	C12—C13	1.336 (4)
C4—C5	1.390 (3)	C13—C14	1.342 (5)
C5—C6	1.371 (3)	C13—H13	0.9300
C5—H5	0.9300	C14—C15	1.384 (4)
C6—C7	1.365 (3)	C15—H15	0.9300
			
C4—O2—C9	118.86 (17)	C7—C8—H8	118.6
C2—C1—H1A	109.5	C3—C8—H8	118.6
C2—C1—H1B	109.5	O2—C9—C10	106.94 (18)
H1A—C1—H1B	109.5	O2—C9—H9A	110.3
C2—C1—H1C	109.5	C10—C9—H9A	110.3
H1A—C1—H1C	109.5	O2—C9—H9B	110.3
H1B—C1—H1C	109.5	C10—C9—H9B	110.3
O1—C2—C1	119.4 (2)	H9A—C9—H9B	108.6
O1—C2—C3	118.1 (2)	C11—C10—C15	118.4 (2)
C1—C2—C3	122.6 (2)	C11—C10—C9	119.5 (2)
C8—C3—C4	116.9 (2)	C15—C10—C9	122.0 (3)
C8—C3—C2	117.04 (19)	C12—C11—C10	119.4 (3)
C4—C3—C2	126.04 (19)	C12—C11—H11	120.3
O2—C4—C5	122.9 (2)	C10—C11—H11	120.3
O2—C4—C3	116.96 (18)	C13—C12—F1	118.0 (3)
C5—C4—C3	120.2 (2)	C13—C12—C11	123.8 (3)
C6—C5—C4	120.2 (2)	F1—C12—C11	118.2 (3)
C6—C5—H5	119.9	C12—C13—C14	116.5 (3)
C4—C5—H5	119.9	C12—C13—H13	121.7
C7—C6—C5	120.9 (2)	C14—C13—H13	121.7
C7—C6—H6	119.6	F2—C14—C13	118.8 (3)
C5—C6—H6	119.6	F2—C14—C15	118.6 (4)
C6—C7—C8	118.9 (2)	C13—C14—C15	122.6 (3)
C6—C7—H7	120.5	C10—C15—C14	119.2 (3)
C8—C7—H7	120.5	C10—C15—H15	120.4
C7—C8—C3	122.9 (2)	C14—C15—H15	120.4
			
O1—C2—C3—C8	−1.7 (3)	C2—C3—C8—C7	179.8 (2)
C1—C2—C3—C8	178.3 (2)	C4—O2—C9—C10	−173.0 (2)
O1—C2—C3—C4	178.8 (2)	O2—C9—C10—C11	107.6 (3)
C1—C2—C3—C4	−1.2 (3)	O2—C9—C10—C15	−70.1 (3)
C9—O2—C4—C5	0.1 (3)	C15—C10—C11—C12	−0.2 (4)
C9—O2—C4—C3	179.2 (2)	C9—C10—C11—C12	−177.9 (2)
C8—C3—C4—O2	−178.31 (19)	C10—C11—C12—C13	1.0 (5)
C2—C3—C4—O2	1.2 (3)	C10—C11—C12—F1	179.0 (3)
C8—C3—C4—C5	0.8 (3)	F1—C12—C13—C14	−179.2 (3)
C2—C3—C4—C5	−179.7 (2)	C11—C12—C13—C14	−1.1 (5)
O2—C4—C5—C6	178.5 (2)	C12—C13—C14—F2	179.7 (3)
C3—C4—C5—C6	−0.6 (3)	C12—C13—C14—C15	0.6 (5)
C4—C5—C6—C7	0.1 (4)	C11—C10—C15—C14	−0.3 (4)
C5—C6—C7—C8	0.1 (4)	C9—C10—C15—C14	177.3 (3)
C6—C7—C8—C3	0.2 (4)	F2—C14—C15—C10	−179.0 (3)
C4—C3—C8—C7	−0.6 (3)	C13—C14—C15—C10	0.1 (5)
